# Causal Effects of Atrial Fibrillation and Warfarin Use on Osteoporosis Risk: A Two‐Sample Mendelian Randomization Analysis

**DOI:** 10.1155/ijog/7863262

**Published:** 2026-04-20

**Authors:** Xiao Hu, Jiaqin Cai, Xiaoxia Wei, Hong Sun

**Affiliations:** ^1^ Department of Pharmacy, Fuzhou University Affiliated Provincial Hospital, Fuzhou, 350000, Fujian, China; ^2^ Department of Pharmacy, Shengli Clinical Medical College of Fujian Medical University, Fuzhou, 350000, Fujian, China, fjmu.edu.cn

**Keywords:** atrial fibrillation, bone mineral density, causal inference, genome-wide association study, Mendelian randomisation, osteoporosis, vitamin K antagonists, warfarin

## Abstract

**Objective:**

A number of observational studies have previously reported an association between atrial fibrillation (AF), long‐term warfarin therapy, and an increased risk of osteoporosis (OP). The proposed mechanisms behind this association involve chronic inflammation or vitamin K‐dependent bone metabolism. However, the presence of confounding factors and methodological limitations precludes the drawing of causal conclusions. The present Mendelian randomisation (MR) study investigates the existence of genetic evidence for causal links between AF, warfarin use and OP development.

**Methods:**

The two‐sample MR was performed using summary statistics from European‐ancestry genome‐wide association studies. Genetic instruments for AF (111 independent SNPs) and warfarin use (9 SNPs) were selected at stringent significance thresholds (*p* < 5 × 10^−8^). The OP dataset comprised 7751 cases and 476,847 controls from UK Biobank. The primary analyses employed inverse‐variance‐weighted (IVW) regression, supplemented by MR‐Egger, weighted median methods, and sensitivity analyses evaluating pleiotropy and heterogeneity.

**Results:**

IVW analysis showed no causal association between genetic predisposition to AF and OP risk (OR = 1.0006, 95% CI 0.9998–1.0014, *p* = 0.114), with the extremely tight confidence interval from our large, high‐precision sample ruling out any clinically meaningful effect. In a similar manner, genetically proxied warfarin exposure demonstrated no substantial influence on OP susceptibility (IVW OR = 1.0445, 95% CI: 0.941–1.159, *p* = 0.412). Sensitivity analyses were conducted to assess the stability of the results, and no evidence of horizontal pleiotropy (MR‐Egger intercept *p* > 0.05) or heterogeneity (Cochran’s *Q* *p* > 0.05) was identified. Iterative leave‐one‐out analyses demonstrated that no individual SNP exerted a disproportionate influence on the estimates.

**Conclusions:**

This study, informed by genetic research, challenges established causal relationships between AF, warfarin use and the risk of OP. The observed clinical associations may be attributable to residual confounding or comorbidities present in ageing populations, rather than direct pharmacological effects. These findings provide a scientific basis for the clinical prioritisation of the management of thromboembolic risk over speculative concerns regarding bone health in the treatment of AF.

## 1. Introduction

Osteoporosis (OP) is a systemic skeletal disorder characterised by compromised bone microarchitecture and fragility fractures. It predominantly manifests in two forms: The first is characterised by oestrogen deficiency (postmenopausal), and the second is age‐related (senile). A secondary form of OP may also arise from chronic comorbidities or pharmacological interventions [1, 2]. The epidemiological characteristics of OP demonstrate marked regional variations and demographic disparities. According to the Global Burden of Disease Study (2019), the number of incident cases worldwide was 41.5 million, with projections indicating a rise to 52.5 million cases by 2030–2034 [3]. Systematic reviews estimate a global prevalence of 19.7% (95% CI 18.0%–21.4%) [4], with notably high healthcare burdens observed in developed nations. In the United States, approximately 54 million adults currently live with OP, and epidemiological models predict that half of postmenopausal women will experience osteoporotic fractures. By 2025, it is anticipated that the associated direct medical costs will exceed $25 billion [5]. European studies predict that the number of bone disease patients will exceed 30 million by 2050, with the total cost of hospital treatment alone projected to reach €17.7 billion [6]. It is projected that global expenditure on OP management will exceed $25 billion by 2025 [7].

The escalating predominance of OP and population ageing have accentuated the clinical convergence between OP and atrial fibrillation (AF) [8]. Observational studies have demonstrated significant associations between OP and AF, particularly noting increased AF incidence among OP patients [9–11]. The observed association may be attributable to shared pathophysiological mechanisms, particularly chronic low‐grade inflammation. In the pathogenesis of AF, inflammatory cytokines such as interleukin‐6 (IL‐6) and tumour necrosis factor‐alpha (TNF‐α) have been demonstrated to mediate atrial fibrosis through cardiac fibroblast activation [12]. Concurrently, these mediators enhance osteoclast differentiation and bone resorption activity via upregulation of the RANKL/nuclear factor‐kappa B (NF‐κB) signalling pathway in bone metabolism [13]. Nevertheless, the establishment of causal inferences remains challenging, as mechanistic overlaps may be indicative of confounding factors rather than direct interactions. A critical unresolved question is whether AF–OP links stem from shared pathways, such as oxidative stress or inflammation, or secondary effects of anticoagulant therapies (e.g., warfarin) commonly prescribed for AF. Elucidation of these mechanisms is imperative for the optimisation of therapeutic strategies in comorbid populations, particularly in light of the ageing demographic. Further research is required that integrates genetic and pharmacoepidemiological approaches. The objective of this research is to disentangle causality from observational correlations.

Warfarin, a vitamin K antagonist (VKA), has been utilised as a fundamental therapeutic approach for the prevention of stroke and systemic thromboembolism in patients with AF for an extended period [14]. In settings where resources are limited, or in specific clinical scenarios (such as following mechanical heart valve replacement), VKAs remain the preferred anticoagulant due to their unique therapeutic profile [15, 16]. The pathophysiological interplay between warfarin use and the development of OP involves multifaceted mechanisms. Inhibition of vitamin K epoxide reductase (VKOR) by warfarin has been demonstrated to disrupt the regeneration of reduced vitamin K, which is required for the γ‐carboxylation of bone‐specific vitamin K‐dependent proteins (VKDPs). This process is particularly significant for the carboxylation of osteocalcin (OC). This pharmacological interference has been shown to elevate circulating undercarboxylated osteocalcin (ucOC) levels, which has been identified as a biomarker reflecting functional vitamin K deficiency. It has been hypothesised that this may in turn impair bone matrix mineralisation [17–19]. From a mechanistic perspective, the process of γ‐carboxylation facilitates the binding of calcium ions by OC through the formation of a Gla domain. In the context of warfarin‐induced carboxylation defects, there is a reduction in OC’s affinity for hydroxyapatite crystals, consequently impacting its function in regulating the rate of bone mineralisation [20]. From a clinical perspective, longitudinal cohort studies have demonstrated a correlation between chronic warfarin exposure and accelerated trabecular bone loss. This finding is analogous to the skeletal alterations observed in populations with suboptimal dietary intake of vitamin K [21]. Moreover, certain studies have demonstrated that, in comparison with warfarin, non‐vitamin K oral anticoagulants (NOACs) are associated with a considerably diminished risk of any fracture and osteoporotic fractures [9, 22]. However, the epidemiological associations observed between AF–OP and warfarin‐OP necessitate rigorous differentiation of three mechanistic possibilities: direct causal relationships versus concurrent age‐related pathological pathways (e.g., inflammaging‐mediated oxidative stress) versus treatment‐induced cascades (such as polypharmacy‐driven deficiencies in vitamin D/K). In order to mitigate confounding factors inherent to ageing populations—particularly those related to frailty and comorbidities, as well as surveillance bias—there is a need to implement causal inference methodologies. These include, but are not limited to, Mendelian randomisation (MR) using genetic instruments for lifelong anticoagulant exposure. The aim of this is to isolate disease‐specific and iatrogenic effects within complex geriatric pathophysiology.

MR is a statistical method that uses genetic variants as instrumental variables to infer causality between exposures and outcomes, thus avoiding the confounding biases that are inherent in observational studies [23]. Specifically, MR employs single‐nucleotide polymorphisms (SNPs) that exhibit a strong association with the exposure—in this case, AF or warfarin use—to proxy lifelong exposure effects on OP. Two‐sample MR (TSMR) has been shown to enhance robustness by analysing exposure and outcome data from independent genome‐wide association study (GWAS) cohorts, such as those catalogued in open‐access repositories [24]. This approach is designed to minimise reverse causation and environmental confounding, thereby addressing the critical limitations of prior nonrandomised analyses [25]. In this study, we implement TSMR to rigorously evaluate the causal influence of AF or warfarin on OP risk. The utilisation of GWAS‐derived genetic instruments is intended to facilitate the disentanglement of direct pharmacological effects from confounding factors (e.g., ageing and comorbidities) that may result in the artificial inflation of OP‐AF associations in traditional studies. This analysis signifies the inaugural implementation of TSMR in addressing this specific clinical query, thereby providing novel insights into the anticoagulant safety profiles and shared pathophysiology observed in ageing populations.

## 2. Materials and Methods

### 2.1. Data Sources

The present analysis involved the examination of summary‐level GWAS data from three sources, which is available in the IEU OpenGWAS repository. AF (ebi‐a‐GCST006414), warfarin use (ukb‐b‐13248) and OP (ebi‐a‐GCST90038656). The AF dataset comprised 1,030,836 European‐ancestry individuals (60,620 cases; 970,216 controls) from five cohorts (2006–2018): The following research organisations have been involved in the study: UK Biobank, deCODE genetics, Michigan Genomics Initiative, DiscovEHR and AFGen Consortium [26]. The data on OP and warfarin originated from the UK Biobank prospective cohort (2006–2018), comprising 484,598 participants (OP: 7751 cases/476,847 controls) [27] and 462,933 individuals (warfarin: 4623 cases/458,310 controls), respectively.

The cases under investigation met the World Health Organization (WHO) diagnostic criteria for OP, as defined by a femoral neck/lumbar spine BMD T‐score of ≤ −2.5 as determined via DXA. This diagnosis was subsequently validated through a review of fragility fracture records. It is important to note that all datasets excluded non‐European individuals, participants with ambiguous sex identification, or insufficient genotyping quality (heterozygosity outliers). In order to minimise the impact of population stratification bias in MR analyses, European‐ancestry cohorts were prioritised on the basis of the availability of large‐scale GWAS summary statistics with homogeneous genetic architectures. The necessity for ethical approval was deemed non‐applicable in this particular instance, as the study in question involved the analysis of fully anonymised, publicly accessible GWAS summary statistics. The study made use of anonymised individual‐level data, with all original GWAS cohorts obtained with the requisite ethical approval and informed consent during their primary data collection phases. The data sources are outlined in Table 1.

**TABLE 1:** Summary of data sources for warfarin, AF and OP.

Exposure/outcome	GWAS ID	Sample size	Number of SNPs	Control	Consortium
AF	ebi‐a‐GCST006414	1,030,836	33,519,037	970,216	MRC‐IEU
Warfarin	ukb‐b‐13248	462,933	9,851,867	458,310	MRC‐IEU
OP	ebi‐a‐GCST90038656	484,598	9,587,836	476,847	MRC‐IEU

*Note:* OP, osteoporosis.

Abbreviation: AF, atrial fibrillation.

### 2.2. Core Assumptions of MR

The validity of MR relies on three core assumptions [28]. First, the relevance assumption: Genetic instruments must be strongly associated with the exposure (AF or warfarin use). Second, the independence assumption: Instruments must not be associated with any confounders of the exposure‐outcome relationship. Third, the exclusion restriction assumption: Instruments must influence the outcome (OP) only through the exposure, not via alternative pathways [29, 30]. To ensure these assumptions were met, we conducted a series of quality control and sensitivity analyses, as detailed in the following sections.

### 2.3. Genetic Instrument Curation

Genetic instruments for AF and warfarin dose were selected from GWAS summary data using a genome‐wide significance threshold (*p* < 5 × 10^−8^) [31, 32]. In order to minimise pleiotropic effects and ensure variant independence, LD clumping was performed (*r* ^2^ < 0.001, 10,000 kb window) [33]. In the context of the OP GWAS data, the subsequent filtration (MAF ≥ 1%) and harmonisation of the extracted SNPs were undertaken to align exposure‐outcome alleles. In order to prevent directional bias, ambiguous or palindromic SNPs, along with those lacking effect estimates or exhibiting allele mismatches, were excluded from the analysis [34, 35]. To assess the relevance assumption, we calculated F‐statistics for each SNP using the formula *F* = β^2^_exposure/SE^2^_exposure; all SNPs exceeded the conventional threshold of 10, indicating strong instruments and minimal weak instrument bias [36]. This rigorous protocol serves to enhance the validity of the instruments in question by addressing such concerns as linkage disequilibrium, population stratification and horizontal pleiotropy. These issues represent significant challenges to the assumptions underpinning MR. The integration of exposure‐specific genetic variants with outcome data, undertaken with rigorous quality control measures, enhances causal inference robustness in comparison to prior studies that inadequately addressed these confounders.

### 2.4. Causal Relationship Verification Methods

Five MR approaches were employed to assess causal effects of AF and warfarin use on OP: inverse‐variance‐weighted (IVW), MR‐Egger, weighted median, simple mode and weighted mode. IVW functioned as the primary estimator, utilising a weighting scheme based on the inverse variance to calculate SNP‐exposure effects [37]. MR‐Egger regression was used to evaluate horizontal pleiotropy via intercept testing, with an intercept *p* > 0.05 considered indicative of no significant pleiotropy; this directly addresses the exclusion restriction assumption [38]. The weighted median provided robustness against invalid instruments under the majority‐valid assumption [39]. Mode‐based methods were utilised to identify modal causal estimates across SNP clusters. Heterogeneity was quantified using Cochran’s *Q* statistic; when detected (*Q* *p* < 0.05), random‐effects IVW was prioritised over fixed‐effects models [40]. The stability of the results was tested by means of leave‐one‐out sensitivity analyses, whereby individual SNPs were iteratively excluded [41]. This multimethod framework enhances the reliability of causal inference in comparison to single‐model approaches whilst transparently acknowledging limitations in instrument diversity and subgroup analyses.

However, stratified analyses (e.g., sex‐specific effects) and bidirectional MR were omitted due to limited instrument counts (AF: 111 SNPs; warfarin: 9 SNPs). This compromise in statistical power would have a detrimental effect on the subgroup or reverse‐causality frameworks. It is recommended that future studies utilise expanded GWAS cohorts in order to address the identified gaps in the current research. All analyses were conducted in R 4.4.1 with the TwoSampleMR package at *α* = 0.05.

### 2.5. Data Availability

GWAS summary statistics for AF (GWAS ID: ebi‐a‐GCST006414), warfarin use (GWAS ID: ukb‐b‐13248) and OP (GWAS ID: ebi‐a‐GCST90038656) are accessible to the public via the IEU OpenGWAS repository (https://gwas.mrcieu.ac.uk/). The R scripts for MR analyses can be obtained from the corresponding author upon reasonable request.

## 3. Results

### 3.1. Genetic Instrument Selection for AF/Warfarin and OP Associations

In this study, we identified SNPs with strong association characteristics (*p* < 0.05) between OP and AF, or OP and warfarin, from the TSMR analysis. Following the exclusion of SNPs that did not yield relevant outcomes and the adjustment or deletion of palindromic SNPs, a total of 111 SNPs were included in the MR analysis of AF and OP and 9 SNPs were included in the MR analysis of warfarin and OP. Following a comprehensive review of the relevant literature, it was determined that a total of 120 SNPs would be utilised for the TSMR analysis. The details of these genetic instruments, including their effect alleles and association statistics, are documented in Supporting Tables 1 and 2. Supporting Table 1 contains the details for AF, and Supporting Table 2 contains the details for warfarin use.

**FIGURE 1:**
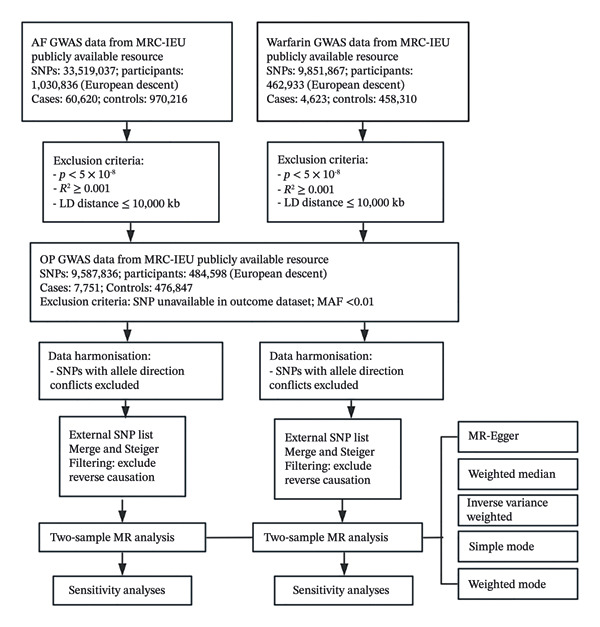
Flowchart of GWAS data processing for AF and warfarin in relation to OP. Note: GWAS, genome‐wide association study; AF, atrial fibrillation; OP, osteoporosis.

The data processing flowchart for the GWAS investigating the associations between AF/warfarin and OP is presented in Figure 1.

### 3.2. Two‐Sample MR Analyses

The IVW analysis revealed no evidence of causal effects of AF on OP risk (OR = 1.0006, 95% CI: 0.9998–1.0014; *p* = 0.114). In a similar manner, the utilisation of genetically proxied warfarin revealed no correlation with OP susceptibility (OR = 1.0445, 95% CI: 0.941–1.159; *p* = 0.412) in the primary IVW model. The complete results from the complementary MR methods (MR‐Egger, weighted median) are systematically presented in Table 2, demonstrating consistent null associations across sensitivity analyses.

**TABLE 2:** TSMR analysis results of AF/warfarin and OP.

Exposure/outcome	Method	SNP (*n*)	OR (95% CI)	*p* value
AF/OP	MR‐Egger	111	1.0008 (0.9993–1.0024)	0.3044
	Weighted median	111	1.0006 (0.9992–1.0021)	0.3939
	Inverse‐variance‐weighted	111	1.0006 (0.9998–1.0014)	0.1144
	Simple mode	111	1.0003 (0.9975–1.0032)	0.8249
	Weighted mode	111	1.0005 (0.9990–1.0020)	0.5161

Warfarin/OP	MR‐Egger	9	1.1590 (0.8767–1.5321)	0.3347
	Weighted median	9	1.0637 (0.9287–1.2183)	0.3724
	Inverse‐variance‐weighted	9	1.0445 (0.9413–1.1589)	0.4123
	Simple mode	9	1.0921 (0.8871–1.3446)	0.4303
	Weighted mode	9	1.0681 (0.9166–1.2448)	0.4231

*Note:* OP, osteoporosis.

Abbreviation: AF, atrial fibrillation.

### 3.3. Sensitivity Analyses

In conducting MR analysis to assess the relationship between AF/warfarin and OP, heterogeneity tests, pleiotropy tests and leave‐one‐out analyses were employed to examine the robustness and reliability of the results.

#### 3.3.1. AF and OP

MR analyses for AF revealed no significant heterogeneity across instrumental SNPs, with Cochran’s *Q* statistics of 131.08 (*p* = 0.074) for MR‐Egger and 131.16 (*p* = 0.083) for IVW (see Figure 2(a)). Figure 2(b), the forest plot, visually represents the ORs and 95% CIs for individual SNPs. It further supports the absence of a causal effect of AF on OP risk and reinforces the robustness of the primary findings. Leave‐one‐out sensitivity analyses (Figure 2(c)) confirmed the stability of the model, with no single SNP exerting a disproportionate influence on causal estimates. The absence of horizontal pleiotropy was evidenced by a nonsignificant MR‐Egger intercept (*β* = −1.65 × 10^−5^, *p* = 0.791) and symmetrical funnel plot distributions (Figure 2(d)). These findings provide robust evidence to refute the hypothesis of a causal relationship between AF and OP risk.

FIGURE 2Sensitivity analyses of Mendelian randomisation estimates for AF on OP risk. (a–d) Scatter plots (a), forest plots (b), leave‐one‐out plots (c) and funnel plots (d) of causal effect estimates. Note: AF, atrial fibrillation; OP, osteoporosis.

**Figure:**
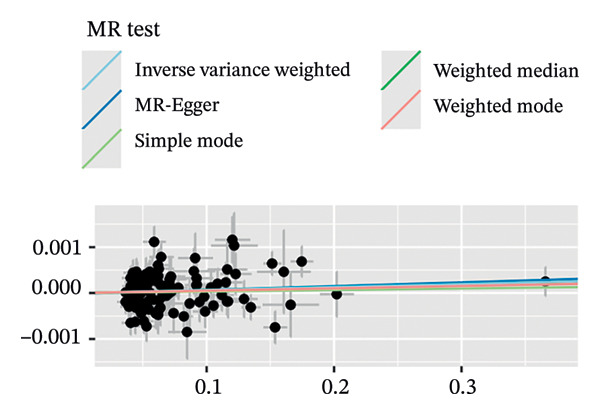
(a)

**Figure:**
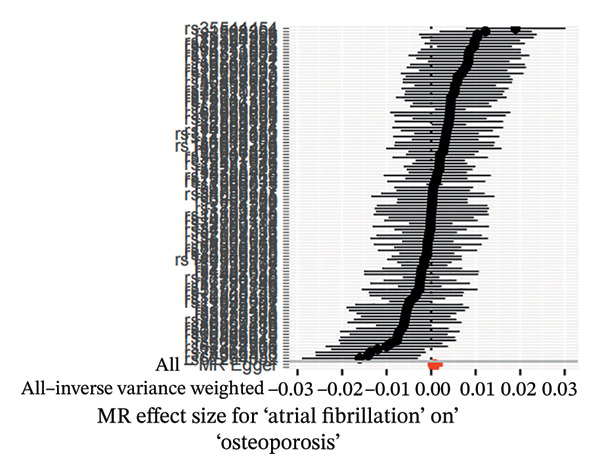
(b)

**Figure:**
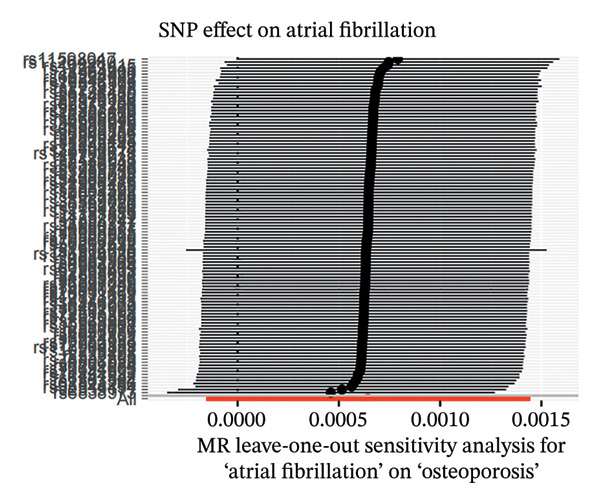
(c)

**Figure:**
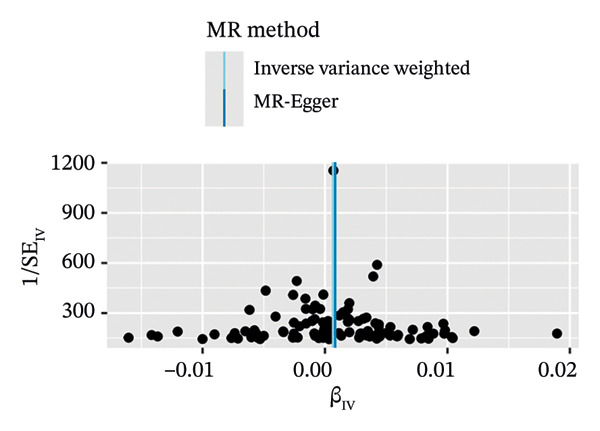
(d)

#### 3.3.2. Warfarin Use and OP

In a similar manner, warfarin‐associated instruments demonstrated minimal heterogeneity (MR‐Egger *Q* = 7.81, *p* = 0.350; IVW *Q* = 8.50, *p* = 0.386) and no indication of pleiotropy (intercept *β* = −0.00023, *p* = 0.455; Figure 3(a)). Figure 3(b), the forest plot, visually represents the ORs and 95% CIs for individual SNPs. It further supports the absence of a causal effect of warfarin on OP risk and reinforces the robustness of the primary findings. The application of leave‐one‐out analyses (Figure 3(c)) revealed that the exclusion of individual SNPs resulted in the preservation of null associations, with all causal estimates falling within the confidence intervals. The absence of horizontal pleiotropy was evidenced by a nonsignificant MR‐Egger intercept (*β* = −22.94 × 10^−5^, *p* = 0.455) and symmetrical funnel plot distributions (Figure 3(d)). This consistency across sensitivity frameworks—spanning pleiotropy tests, heterogeneity assessments and outlier detection—reinforces the absence of a direct causal effect of warfarin on OP.

FIGURE 3Sensitivity analyses of Mendelian randomisation estimates for warfarin use on OP risk. (a–d) Scatter plots (a), forest plots (b), leave‐one‐out plots (c) and funnel plots (d) of causal effect estimates. Note: AF, atrial fibrillation; OP, osteoporosis.

**Figure:**
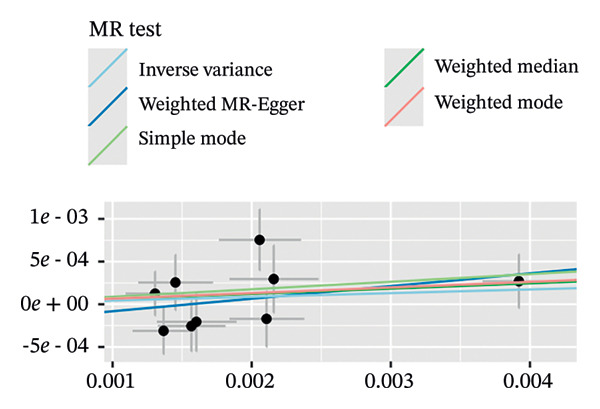
(a)

**Figure:**
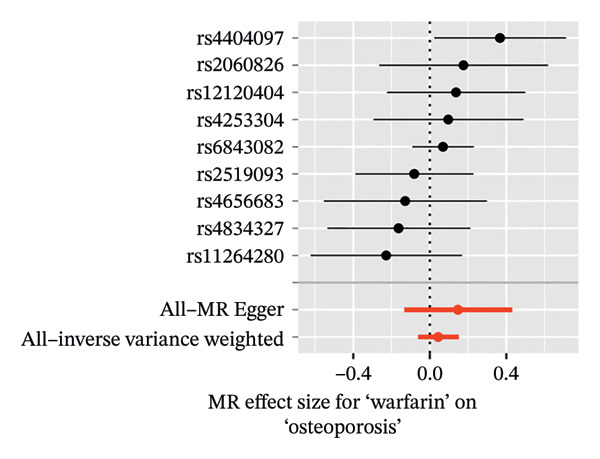
(b)

**Figure:**
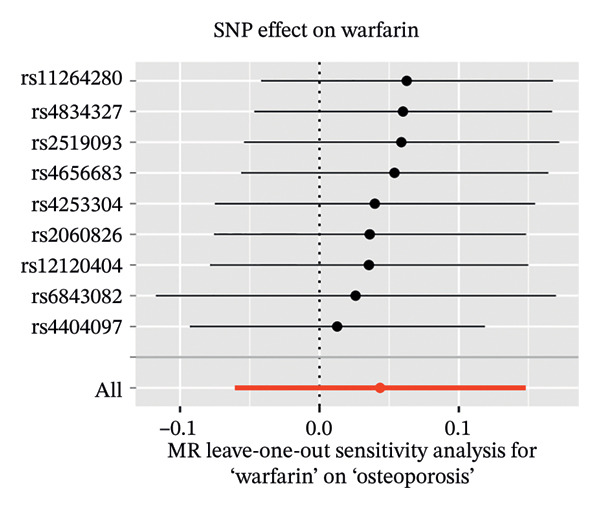
(c)

**Figure:**
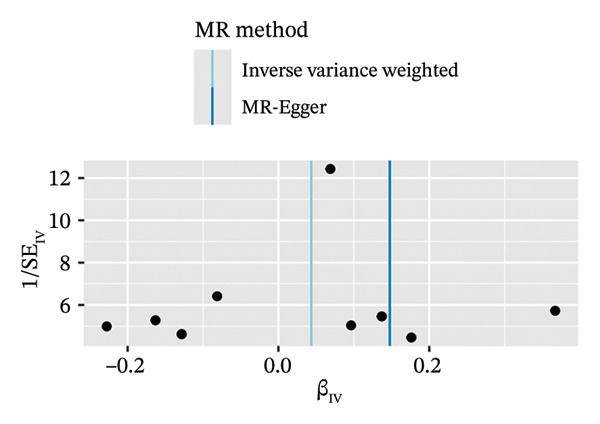
(d)

The collective outcome of these sensitivity analyses serves to reinforce the robustness of the null findings. Future investigations employing ancestry‐diverse cohorts, expanded SNP panels, or nonlinear MR approaches could further elucidate these relationships.

## 4. Discussion

The MR analysis provides robust genetic evidence against a causal relationship between AF, warfarin use and OP risk. The null associations were observed to persist across a range of sensitivity frameworks, including pleiotropy‐robust MR‐Egger regression and leave‐one‐out analyses (see Figures 2(c) and 3(c)). The odds ratios were found to be closely concentrated around the null value (AF–OP: OR = 1.0006, 95% CI = 0.9998–1.0014; warfarin‐OP: OR = 1.0445, 95% CI = 0.9413–1.1589). Particularly noteworthy is the precision of the IVW estimate for the effect of AF on OP, which is extremely close to the null and exhibits a very tight confidence interval. This precise estimate suggests that even if a causal effect exists, it is likely to be so small as to be clinically irrelevant. This precision is a key strength of our study, as the large‐scale GWAS data provide sufficient power to rule out even a modest effect. This finding further bolsters the argument against routine BMD monitoring specifically for AF patients without other risk factors. These findings call into question the conclusions of observational studies that have previously reported an elevated fracture risk in warfarin users. This discrepancy may be attributed to residual confounding in observational studies. This bias is addressed by MR’s quasi‐randomised design. It is noteworthy that while biochemical studies have demonstrated that VKAs impair OC carboxylation [17–19], the present results suggest that compensatory mechanisms (e.g., Wnt/β‐catenin upregulation) or variations in dietary vitamin K intake may counteract these effects in vivo [42, 43]. In the context of warfarin, our null genetic findings extend the scope of RCT meta‐analyses by addressing time‐related biases that are inherent to observational pharmacoepidemiology. For instance, although retrospective cohorts have been shown to demonstrate an association between long‐term warfarin use and fracture risk, such analyses often fail to adequately adjust for cumulative comorbidities or surveillance bias. MR circumvents these issues by proxying lifelong warfarin exposure through genetic instruments, thus isolating pharmacological effects from confounding by indication. This distinction highlights the necessity of differentiating between drug use patterns (e.g., duration and adherence) and drug class effects in causal inference.

The findings of this study directly impact the field of AF management. Firstly, the necessity for routine bone mineral density monitoring in warfarin users without additional OP risk factors (e.g., prior fractures and glucocorticoid use) is refuted. Instead, clinicians should prioritise modifiable risks such as physical inactivity, vitamin D deficiency, and smoking cessation in strategies aimed at the prevention of OP. Secondly, the absence of a causal relationship between warfarin and OP supports the implementation of deprescribing initiatives that avoid unnecessary restrictions on the choice of anticoagulants. In making therapeutic decisions, particular emphasis should be placed on thromboembolic risk, bleeding propensity and patient preferences, with these considerations taking precedence over unsubstantiated concerns regarding bone health. For instance, in elderly AF patients with high fall risk but low fracture susceptibility, this finding reinforces that the fear of OP should not be a deciding factor against warfarin when it is otherwise the clinically appropriate or necessary choice.

A significant strength of this study is the utilisation of large‐scale GWAS data to minimise weak instrument bias, thereby ensuring robust SNP‐exposure associations [44]. The TSMR framework has been developed to further mitigate confounding by leveraging genetic variants randomly allocated at conception, thereby effectively emulating a randomised trial [45]. Nevertheless, it is imperative to acknowledge the limitations that are associated with this approach. Firstly, although the analysis was conducted using large‐scale European‐ancestry cohorts in order to maximise genetic homogeneity, the lack of trans‐ethnic replication (e.g., Asian or African populations) limits the generalisability of the results. Secondly, there is a possibility that unmeasured confounding factors may yet be identified as a potential source of bias in the results. Thirdly, the dichotomous classification of warfarin exposure in GWAS data limits dose–response assessments. While the MR analysis examined the general impact of warfarin use versus non‐use, it could not evaluate whether extended duration or higher doses increase skeletal risks. This is an important limitation given the observational evidence associating long‐term warfarin treatment (≥ 1 year) with accelerated bone mineral density decline and fracture incidence [46]. It is also worth noting that the MR design inherently tests for a linear relationship, whereas the observational literature often focuses on long‐term warfarin use as a specific risk factor. The binary nature of the warfarin exposure GWAS cannot distinguish between short‐term and long‐term users, which may obscure risk patterns that only emerge after prolonged exposure.

Fourthly, while the AF and OP data were obtained from independent consortia, it is worth noting that the OP data and warfarin exposure data were both derived from the UK Biobank. Although two‐sample MR does not strictly require completely non‐overlapping samples, sample overlap can introduce bias. We have calculated F‐statistics to assess instrument strength, which helps to mitigate this concern; nonetheless, this partial sample overlap remains a point to consider when interpreting the findings.

In subsequent research, several avenues merit prioritisation. Firstly, multi‐ethnic replication: The validation of these findings in ancestrally diverse cohorts (e.g., All of Us, UK Biobank) will elucidate whether genetic or environmental modifiers (e.g., vitamin K‐rich diets in Mediterranean populations) modulate warfarin‐OP associations. Secondly, to address the limitations of binary warfarin exposure classification and the linearity assumption inherent in MR, future studies should pursue two complementary approaches: (1) employing nonlinear MR methods or analysing time‐to‐event data within a one‐sample MR framework to determine whether a threshold effect exists, where skeletal risk only manifests after many years of treatment and (2) refining exposure characterisation through the integration of drug duration and dosage data to capture potential cumulative skeletal effects in long‐term users. Such approaches would provide a more complete answer to the clinical question regarding long‐term warfarin safety. Additionally, studies using fully independent samples would further strengthen the evidence regarding sample overlap.

## 5. Conclusions

This MR study provides high‐quality genetic evidence against causal effects of AF or warfarin on OP risk. By addressing confounding biases that are pervasive in observational research, the findings recalibrate clinical priorities in the management of AF, advocating for anticoagulant strategies focused on thromboembolic prevention rather than speculative bone health trade‐offs. Subsequent studies that integrate pharmacogenomic and longitudinal real‐world data will further elucidate the interplay between anticoagulation, bone metabolism and ageing.

## Author Contributions

Xiao Hu and Hong Sun conceived and designed the study;

Xiao Hu, Jiaqin Cai and Xiaoxia Wei collected the data;

Xiao Hu, Jiaqin Cai and Xiaoxia Wei are analysed and interpreted the data;

Xiao Hu wrote the manuscript;

Xiao Hu and Hong Sun provided critical revisions that are important for the intellectual content.

## Funding

This research was sponsored by Fujian Provincial Health Technology Project (No. 2023TG003) and the Research Project on High Quality Development of Hospital Pharmacy, National Institute of Hospital Administration, NHC, China (No. NIHAYS2409).

## Disclosure

All authors approved the final version of the manuscript.

## Ethics Statement

Not applicable, because GWAS belongs to public databases; the patients involved in the database have obtained ethical approval; users can download relevant data for free for research and publish relevant articles; and our study is based on open‐source data, and the Fuzhou University Affiliated Provincial Hospital does not require research using publicly available data to be submitted for review to their ethics committee, so there are no ethical issues.

## Consent

This study did not reveal any personal information of patients.

## Conflicts of Interest

The authors declare no conflicts of interest.

## Supporting Information

Additional supporting information can be found online in the Supporting Information section.

The supporting information for this article can be found in Supporting Table 1 and Supporting Table 2.

## Supporting information


**Supporting Information 1** Supporting Table 1. Atrial fibrillation SNPs for TSMR analysis.

**Supporting Information 2** Supporting Table 2. Warfarin SNPs for TSMR analysis.

## Data Availability

Genome‐wide association study summary statistics for atrial fibrillation (AF; GWAS ID: ebi‐a‐GCST006414), warfarin use (GWAS ID: ukb‐b‐13248) and osteoporosis (OP; GWAS ID: ebi‐a‐GCST90038656) are accessible to the public via the IEU OpenGWAS repository (https://gwas.mrcieu.ac.uk/). The R scripts for MR analyses can be obtained from the corresponding author upon reasonable request.
